# Sinensetin attenuates hepatic ischemia-reperfusion injury through suppressing GRP78/CHOP-mediated endoplasmic reticulum (ER) stress in mice

**DOI:** 10.3389/fphar.2025.1519497

**Published:** 2025-02-12

**Authors:** Yihong Yang, Guanghua Xiong, Huangqi Shi, Yulin Peng, Jinghong Liu, Yaxuan Jiang, Min Lu, Hongbo Liu, Yong Liu

**Affiliations:** ^1^ Emergency Department of Fuyang People’s Hospital, Fuyang, Anhui, China; ^2^ Key Laboratory of Embryo Development and Reproductive Regulation of Anhui Province, College of Biology and Food Engineering, Fuyang Normal University, Fuyang, Anhui, China

**Keywords:** sinensetin, HIRI injury, ER stress, liver apoptosis, oxidative stress, 4-PBA

## Abstract

**Objective:**

Hepatic ischemia-reperfusion injury (HIRI) frequently occurs as a complication in liver surgeries, which significantly impacting patient outcomes. Sinensetin (SEN) is a plant-derived polymethoxylated flavone with anti-inflammatory and anti-oxidative activities. However, the hepatoprotective effect of sinensetin in HIRI pathogenesis have not been fully explored.

**Methods:**

We constructed the HIRI model in mice, with blood and liver samples collected at 6 and 24 h after reperfusion to evaluate liver injury. We also evaluated the protective effect of sinensetin in mice liver I/R injury through histopathological observation, enzyme activity, immunofluorescence, Western blot, molecular docking, and molecular pharmacology experiments.

**Results:**

In our study, we have successfully established the mouse HIRI injury model, and the liver function indicators such as ALT, AST and LDH were significantly increased in the HIRI model group, while SEN pretreatment could lead to a significant decrease in these enzymatic activities, especially perfusion at 6 h. In addition, hepatocytic necrosis and lipid deposition were significantly improved under SEN pretreatment conditions compared to the HIRI group alone. Meanwhile, HIRI can significantly increase the expression of genes related to liver injury and inflammation, while SEN pretreatment can lead to a concentration-dependent decrease in these genes. Besides, the level of liver apoptosis and apoptosis-related genes such as BAX and Bcl-2 were significantly reduced especially in the high concentration SEN pretreatment group, and antioxidant enzyme activities such as CAT and GSH-Px also showed similar changes. Moreover, the HIRI model and SEN pretreatment could lead to dynamic changes in key genes involved in endoplasmic reticulum (ER) stress signaling, while the expression and distribution of GRP78 and CHOP proteins in liver cells also showed significant decrease in HIRI + L-SEN and HIRI + H-SEN groups. Molecular docking simulation showed theoretical binding between SEN-GRP78 and SEN-IRE1α in three-dimensional structures. Ultimately, the use of 4-PBA to pharmacologically inhibit ER stress may substantially reduce liver damage caused by HIRI in mice.

**Conclusion:**

Taken together, our results suggested that sinensetin could alleviate HIRI injury through suppressing GRP78/CHOP-mediated ER stress, which may provide a novel therapeutic strategy for treating liver ischemia-reperfusion injury in clinical practice.

## 1 Introduction

Hepatic ischemia/reperfusion injury (HIRI) is a significant clinical challenge that occurs during liver surgeries, such as partial hepatectomy and liver transplantation (Liu et al., 2024; Tan et al., 2024). The HIRI process is characterized by a complex interplay of cellular and molecular mechanisms that lead to liver damage upon the restoration of blood flow after a period of ischemia (Li et al., 2021; Wang et al., 2021; Mao et al., 2023). The pathophysiology of HIRI involves a cascade of inflammatory responses, oxidative stress, and apoptosis, which can ultimately result in hepatocyte necrosis and dysfunction and significantly impact the prognosis of patients (Zhang et al., 2016; Wang et al., 2021). Previous studies have shown that liver reperfusion injury can be divided into two main stages: the acute phase, from the beginning of reperfusion to 6 h, characterized by oxidative stress and inflammatory cytokine induced hepatocyte apoptosis and necrosis (Yang et al., 2016; Gao et al., 2022); The second stage is mainly chronic damage, involving the fibrosis process of liver cells (Li et al., 2015; Chullo et al., 2024). Currently, the treatment drugs for liver ischemia-reperfusion injury include antioxidants such as N-acetylcysteine and superoxide dismutase (SOD), as well as drugs that alleviate ischemic injury such as levorotatory sugar and glycine (Xu et al., 2024). However, these drugs may have some negative effects such as allergic reactions, liver and kidney dysfunction, gastrointestinal discomfort, etc (Krzywonos-Zawadzka et al., 2017). Therefore, understanding the potential pathophysiological mechanisms of HIRI and identifying effective treatment strategies are urgent issues that need to be addressed.

The fruits that are high in phytochemicals has been clearly demonstrated to possess both nutritional and therapeutic benefits, offering possibilities for preventing a range of diseases, such as liver damage (Direito et al., 2024; Fitzgerald et al., 2024). Sinensetin is a polymethoxylated flavonoid found abundantly in citrus fruits and almost harmless to the human body, which possess a number of pharmacological properties such as anti-inflammatory, anti-oxidative and anti-tumor (Li et al., 2020; Zhi et al., 2021). Previous studies have found that many natural drugs play a protective role in liver I/R injury. For example, cordycepin pretreatment ameliorated hepatocyte injury caused by HIRI, which inhibited apoptosis, autophagy and inflammatory response via regulation of the MAPK/NF-κB signaling pathway (Ding et al., 2022). Celastrol protected against cerebral I/R injury through inhibiting glycolysis via the HIF-1α/PDK1 axis (Chen et al., 2022). The ellagic acid is a polyphenolic compound that could ameliorate hepatic I/R injury by inhibiting pyroptosis and the Caspase-1/GSDMD axis (Wang et al., 2022). Melatonin inhibited the NF-κB signaling pathway, alleviated the inflammatory response, and protected the liver from ischemia-reperfusion injury in rats (Gao et al., 2021). However, the potential protective effects of sinensetin against liver ischemia-reperfusion (I/R) injury in mice remains to be elucidated.

During the process of liver ischemia-reperfusion conditions, the large amount of ROS produced after reperfusion and could cause endoplasmic reticulum (ER) stress (Zhou et al., 2016; Zhang et al., 2023). ER stress arises when the balance between protein synthesis, folding, and transport is disrupted *in vivo* (Liu et al., 2012). In hepatic I/R injury, the accumulation of unfolded or misfolded proteins within the ER lumen activates ER stress and these sensors include PERK, IRE1α and ATF6 (Ajoolabady et al., 2023; Chen et al., 2023). GRP78 serves as a chaperone protein and signaling regulator for the endoplasmic reticulum, while CHOP protein acts as a key transcription factor in stress response, playing a crucial role in maintaining cellular homeostasis and combating ER stress (Arab et al., 2023; Ding et al., 2024). Previous studies found that C/EBPα overexpression ameliorated IR-induced hepatic injury, manifesting with reduced ALT/AST, oxidative stress and ER stress (Li et al., 2024). Salidroside attenuates myocardial ischemia/reperfusion injury via AMPK-induced ER stress inhibition (Tian et al., 2022). Dexmedetomidine attenuates hepatic ischemia-reperfusion injury-induced apoptosis via reducing oxidative stress and ER stress (Zhang et al., 2023). However, whether ER stress signaling is involved in the protective effect of sinensetin against HIRI-induced liver injury is largely unknown.

Therefore, we constructed the hepatic ischemia-reperfusion injury model in mice, and we revealed that sinensetin pretreatment provided the significantly protective effect in liver I/R injury of mice by molecular experiments. In addition, we demonstrated that sinensetin could greatly inhibit ER stress in HIRI model. Overall, these findings provide new therapeutic potential of sinensetin in liver ischemia-reperfusion injury during surgical procedures and improving patient outcomes.

## 2 Materials and methods

### 2.1 Drugs and reagents

Sinensetin (Cas no. 2306-27-6) was purchased from ChemFaces Bio-Technology Co., Ltd. (Wuhan, China). The enzyme activity detection kits of alanine aminotransferase (ALT, Cas no. C009-2-1), aspartate aminotransferase (AST, Cas no. C010-2-1), lactic dehydrogenase (LDH, Cas no. A020-2-2), catalase (CAT, A007-1-1), glutathione peroxidase (GSH-Px, A005-1-2) were purchased from Nanjing Jiancheng Bioengineering Institute (Nanjing, China). SYBR Green qPCR Mix (Cas no. BL698A) were obtained from Biosharp Life Sciences (Hefei, China). The TUNEL cell apoptosis detection kit (Cas no. M175881-20T) was obtained from Mairuida Technology Co., Ltd. (Beijing, China). Endoplasmic reticulum stress (ERS) inhibitor 4-PBA (Cas no. HY-A0281) was purchased from MedChemExpress Co., Ltd. (MCE, United States).

### 2.2 Experimental animals and study approval

The healthy male ICR/CD-1 mice (n = 60, weighing 18–22 g and aged 6–8 weeks) were obtained from GemPharmatech Biotechnology Co., Ltd. (Nanjing, China). All mice were kept in sterilized cages within a laboratory animal room that met SPF standards, maintained under regulated conditions (a 12 hlight/dark cycle, humidity at 60% ± 10%, and temperature set to 24°C ± 2°C), and provided with unrestricted access to food and water. All mice were acclimated in the animal room for at least 1 week prior to the experiment.

### 2.3 Establishment of the HIRI model

The HIRI animal model was established by nonlethal segmental (70%) hepatic warm ischemia surgery that described in previous studies. Briefly, the mice were deprived of food for 24 h and only access to water prior to this surgery. After that, the mice were anesthetized by 1.25% sodium pentobarbital through an intraperitoneal injection. Next, we cut and opened the abdominal cavity along the center of the mice abdomen with scissors, while carefully isolating the structure of hepatic portal. Then, sterile microarterial clamps were used to block the blood vessels in the left and middle lobes of the liver, immediately causing partial hepatic ischemia. The abdomen was then covered with saline-soaked gauze, and constant temperature heater provided warmth to the mice. After a duration of 60 min, the arterial clamps were removed to restore blood flow and initiate reperfusion for 6 h or 24 h, respectively. Ultimately, the surgical incision in the abdomen was carefully sutured closed, and the mice were maintained at a body temperature of 36.5°C ± 1°C throughout the procedure before being returned to their cages until they fully regained consciousness.

### 2.4 Experimental grouping and drug treatment

The 60 mice were randomly assigned to one of four groups: 1) a sham surgery group (n = 15; the abdominal cavity was opened to separate the first hepatic portal and quickly closed, without HIRI surgery); 2) HIRI group (n = 15; HIRI without sinensetin pre-treatment); 3) HIRI + L-sinensetin group (n = 15; pre-treatment with sinensetin at 25 mg/kg by intraperitoneal injection once daily for 7 days before surgery); 4) HIRI + H-sinensetin group (n = 15; pretreatment with sinensetin at 50 mg/kg by intraperitoneal injection once daily for 7 days before surgery). After surgery and drug treatment, seven to eight mice from each group were randomly chosen at 6 h and 24 h following the start of reperfusion. Both of the blood samples and liver tissues were promptly collected and preserved at −80°C for additional biochemical analyses.

### 2.5 Serum biochemical analysis

Biochemical index analysis were conducted according to our lab’s previous methods with minor modifications (Xiong et al., 2020; Xiong et al., 2024a). Briefly, the blood samples in each group was collected from mice eyeballs and then centrifuged at 4,500 rpm for 10 min to obtain the supernatant. The serum ALT, AST and LDH levels and the antioxidant enzyme activities of CAT and GSH-Px were measured using the test kits of microplate method in Jiancheng Bioengineering Institute according to the manufacturer’s instructions (Nanjing, China). In brief, the collected serum was reacted with a microplate, and placed it into an automatic biochemical analyzer (SpectraMax iD3, MD, United States), and the OD value in each group was detected under the specified wavelength conditions. Each enzyme activity was calculated using a specific formula and expressed as units per litre from three biological replicates (n = 4).

### 2.6 Histopathological analysis

Sections of liver lobe tissues were rapidly submerged and preserved in 4% PFA overnight at 4°C. Following this, the samples underwent dehydration through a series of ethanol and xylene at varying concentrations before being embedded in paraffin. After that, the liver samples were cut into 5 μm-thick sections using the ultra-thin semiautomatic microtome (Leica RM2235, Germany). Next, the slices were dried at 37°C, deparaffinized, rehydrated through a series of xylene-alcohol solution, finally rinsed with deionized water. Then, the samples in each group were performed with hematoxylin and eosin (H&E) staining kit (Solarbio Cas no. G1120, China) and modified Oil Red O staining kit (Solarbio Cas no. G1261, China) according to the manufacturer’s instructions. Furthermore, the stained sections were subsequently examined and captured with the optical microscope (Olympus BX53, Japan).

### 2.7 RNA extraction and qRT-PCR analysis

Total RNA was extracted from liver tissues utilizing the TRIzol reagent (Invitrogen, Cas no. 15596026CN, United States) in accordance with the manufacturer’s guidelines. Subsequently, reverse transcription was carried out and the resulting cDNA was quantified using the SYBR Green PCR Master Mix (Biosharp BL698A, China). The final relative levels of gene expression were determined using the 2^−ΔΔCt^ method. All data were normalized by β-actin and the primer sequences used in this study were shown in Supplementary Table S1.

### 2.8 Immunohistochemical and TUNEL staining analysis

The immunofluorescence staining were performed using the liver tissues in each group according to the previous protocols in our lab (Xiong et al., 2019; Xiong et al., 2024b). Briefly, the samples were dehydrated using a gradient of ethanol. After the antigen retrieval process, the slices were immersed in a sodium citrate solution for 10 min at 95°C. After washed with 1x PBS, the samples were hybridized with 5% bovine serum albumin (BSA), and the slices were probed with primary antibodies against GRP78 and CHOP (1:500 dilution) for overnight. Next day, the samples were incubated with Goat Anti-Rabbit IgG H&L (Alexa Fluor^®^ 488, Abcam ab150077) and Goat Anti-Rabbit IgG H&L (Alexa Fluor 647, Abcam ab150079) for 1 h, respectively. Finally, the sections were counterstained with DAPI for 10 min and observed on the laser-scanning confocal microscope (Leica TCS SP5, Germany). The integrated optical densities and differences of protein expression distribution in each group were analyzed using Image-pro Plus software (Media Cybernetics, United States).

The liver apoptosis in each group was detected with the TUNEL assay. Briefly, the prepared slices were dewaxed, dehydrated, and then digested with 20 μg/mL of protease K. Subsequently, 50 μL of TdT enzyme was incubated with the slices at 37°C for 30 min. Next, the samples were stained with TUNEL fluorescence staining solution in One Step TUNEL Apoptosis Detection Kit (Green, Dye 488, Beyotime Cas no. C1088, China) at 37°C for 1 h after that, the sections were washed with 1 x PBS for three times and observed by using laser-scanning confocal microscope. The representative images were randomly collected from each section and the relative fluorescence intensity was analyzed by ImageJ software.

### 2.9 Western blot analysis

The liver samples were homogenized and lysized in RIPA buffer added with proteinase and phosphatase inhibitor cocktail (Solarbio Cas no. P1264, China). The protein concentration in each sample was determined using a BCA protein quantification kit (Thermo Scientific Cas no. A55865, China). Next, the samples were separated in SDS-PAGE electrophoresis and transferred onto the PVDF membranes. After that, the membranes were blocked with 5% BSA and then incubated with primary antibodies against Bcl-2 (dilution 1:2000, Abcam Cas no. ab182858, United Kingdom), BAX (dilution 1:2000, Abcam Cas no. ab32503, United Kingdom), Caspase3 (dilution 1:2000, Abcam Cas no. ab32499, United Kingdom), GRP78 (dilution 1:2000, Beyotime Cas no. AF0171, China), CHOP (dilution 1:2000, SAB Cas no. 49418, United States), ATF6 (dilution 1:2000, SAB Cas no. 24382, United States), IRE1α (dilution 1:2000, SANTA CRUZ Cas no. sc-390960, United States), phospho-eIF2α (Ser51/Ser52) (dilution 1:2000, Affinity Cas no. AF3087, United States) and GAPDH (dilution 1: 5000, ProteinTech Cas no. 10494-1-AP, China) overnight at 4°C. Subsequently, the membranes were incubated with HRP-conjugated secondary antibody (dilution 1:10,000, ProteinTech, Cas no. SA00001-2, China). In addition, the expression of target proteins was measured by chemiluminescence method and the gray values in each group was quantified using the ImageJ v1.8.0 software.

### 2.10 Molecular docking analysis

The theoretical binding ability of SEN-GRP78 and SEN-IRE1α proteins in spatial structure were performed using the AutoDock 4.2 and AutoDock tools 1.5.7 molecular docking software with default parameters. Briefly, the molecular structure of sinensetin was obtained from NCBI PubChem database (PubChem CID:145659). The three-dimensional of GRP78 (P20029) and IRE1_alpha (Q9EQY0) proteins were retrieved from PDB protein database. After removing all heteroatoms, extra chains, water molecules and ions, the processed protein and small molecule ligand were transferred into AutoDock and perform molecular docking using a semi flexible docking method. The docking box size is 120 × 120 × 120 Å^3^ with a grid spacing of 0.375 Å. The obtained molecular docking results were visualized by the PyMOL software and analyzed by taking the conformation with the lowest occupied molecular energy.

### 2.11 Statistical analysis

The statistical data was presented as mean ± SEM and analyzed with GraphPad Prism 9.3 software. Comparisons of the data across groups were conducted using one-way ANOVA and Student’s t-test. Statistical significance was indicated by *p < 0.05 and **p < 0.01, with comparisons to the control or HIRI group considered statistically significant.

## 3 Results

### 3.1 Sinensetin pretreatment alleviated hepatic ischemia-reperfusion injury in mice

**FIGURE 1:**
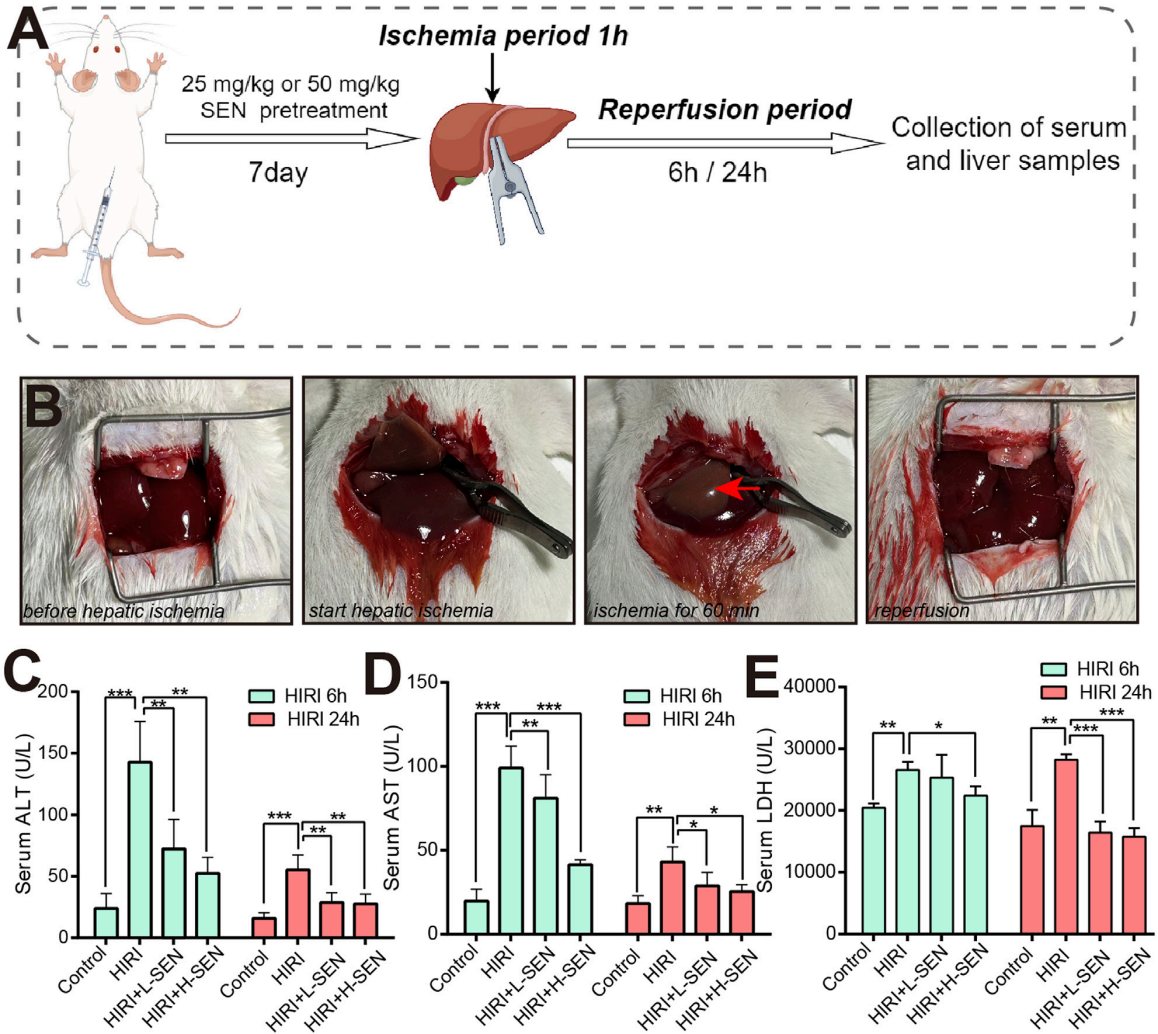
Pre-treatment with sinensetin can significantly alleviate hepatic ischemia/reperfusion injury (HIRI) in mice. **(A)** The schematic diagram showing the construction method of HIRI model and sinensetin drug exposure procedures in mice. **(B)** Representative images of liver morphology at key time points during ischemia-reperfusion process in mice. Red arrow indicated typical features of ischemia in the liver of left lobe. **(C)** The serum ALT levels were presented in sham, HIRI, HIRI + L-SEN and HIRI + H-SEN conditions at 6 h and 24 h, respectively. **(D)** The serum AST levels were measured at HIRI 6 h and HIRI 24 h in each group. **(E)** The serum LDH levels were detected in all treatment groups. The values were presented as mean ± SEM (n = 6, *p < 0.05; **p < 0.01; ***p < 0.001).

To evaluate whether sinensetin plays a protective role in the process of liver HIRI injury, we constructed a mouse HIRI model, and its drug treatment of sinensetin and experimental procedures of HIRI in mice was shown in Figure 1A. It is suggested that the mice hepatic IRI model was established and the representative images of before hepatic ischemia, start hepatic ischemia, warm ischemia for 60 min, and reperfusion were presented in Figure 1B. Our results showed that the arterial clamp significantly occludes vessels in the left and middle lobes of the liver, resulting in immediate hepatic ischemia.

Meanwhile, we analyzed the effects of key liver function indicators at the time points of 6 h and 24 h reperfusion after liver ischemia surgery in mice. When liver cells undergo inflammation or necrosis, alanine transaminase (ALT) will be released into the bloodstream and caused an increase in serum ALT levels. Our results indicated that the enzyme activity of ALT was significantly increased in the HIRI model group, while treatment with sinensetin could significantly reduce its activity level, whether under HIRI + L-SEN or HIRI + H-SEN conditions (Figure 1C). Aspartate transaminase (AST) is mainly present in the mitochondria of liver cells, and when serum AST levels increase, it generally indicates damage to liver cell mitochondria. The results also suggested that AST levels significantly increased in the HIRI model group, while sinensetin showed a concentration-dependent decrease in AST levels under HIRI + L-SEN or HIRI + H-SEN conditions (Figure 1D). It is worth mentioning that the levels of ALT and AST increased more significantly at 6 h of perfusion than at 24 h, suggesting that the cell damage caused by liver ischemia mainly plays a role in the early stages. Lactate dehydrogenase (LDH) is an important enzyme in the process of glycolysis, and an increase in LDH may be related to impaired liver function. Our results found that LDH levels have also increased in the HIRI model group and sinensetin treatment can lead to significant decrease, but unlike ALT or AST, the 24 h of perfusion are more significant than the 6 h, suggesting that this enzyme activity may play a role in the late stage (Figure 1E). Taken together, this results demonstrated that we successfully established a liver ischemia-reperfusion model in mice and preliminarily validated the important protective function of sinensetin during the process of liver ischemia-reperfusion injury in mice.

### 3.2 Sinensetin reduced HIRI-induced liver damage and systemic inflammation

**FIGURE 2:**
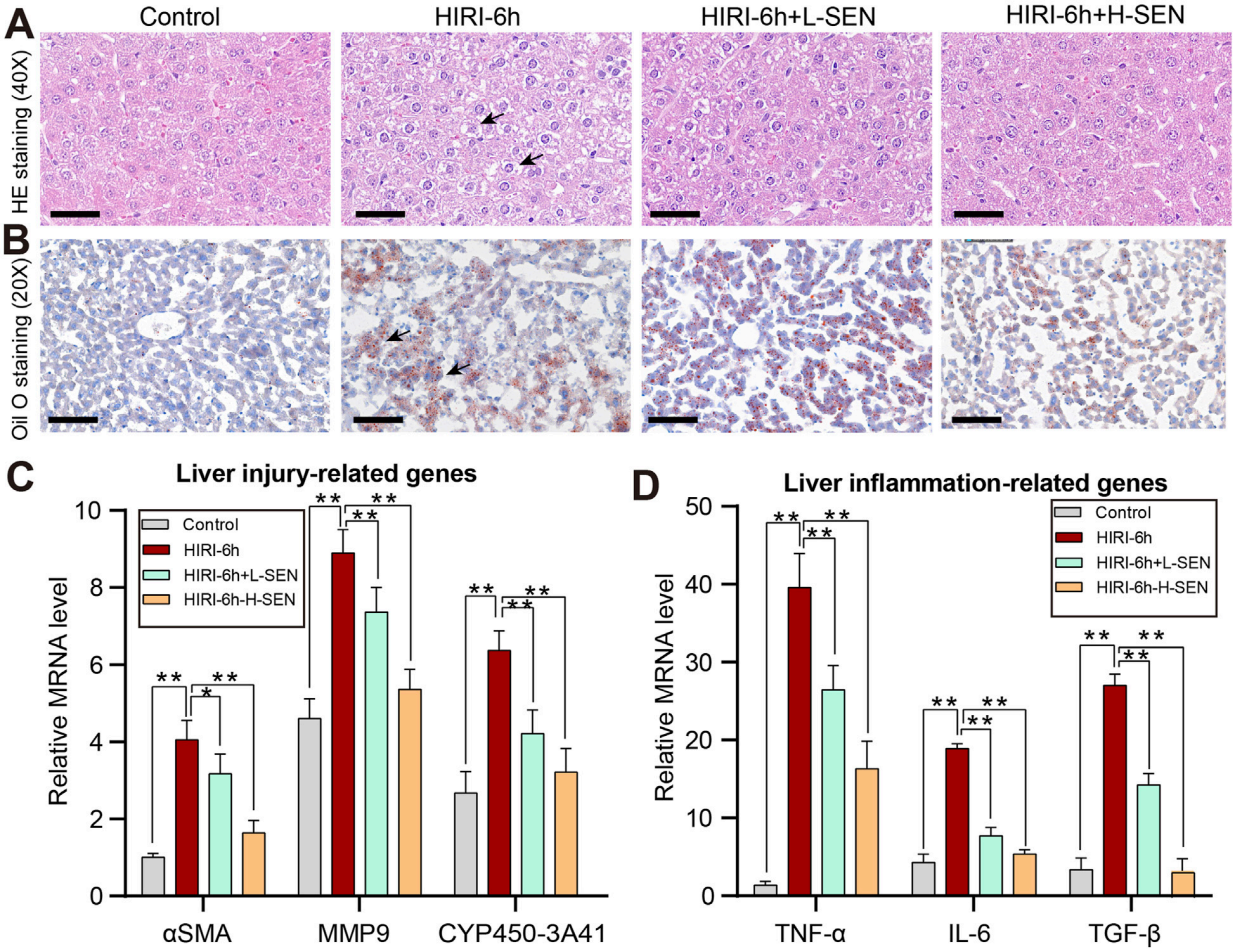
Sinensetin pretreatment could ameliorate HIRI-induced liver damage and inflammatory reaction. **(A)** The liver injury in mice was assessed by hematoxylin and eosin (H&E) staining (original magnification ×200). **(B)** The degree of lipid deposition in mouse liver was analyzed in each group by using Oil Red O staining. **(C)** The expression of liver damage-related genes such as αSMA, MMP9 and CYP450, were analyzed under different conditions by real-time PCR. **(D)** The relative expression levels of inflammatory cytokines in mice liver, including TNF-α, IL-6 and TGF-β, were detected by qRT-PCRs. These results were obtained from at least three independent experiments. Values are presented as mean ± SEM (n = 6). **p < 0.05, **p < 0.01 vs. the HIRI group.

In order to further evaluate the sinensetin pretreatment on the HIRI-induced liver damage, we analyzed the pathological structure and lipid deposition degree of the liver through H&E staining and Oil O staining, respectively. Our results suggested that no liver cell necrosis was observed in the control group, while the HIRI group showed significant hepatocyte necrosis and inflammatory cell infiltration (Figure 2A). Compared with the HIRI group, sinensetin pretreatment can significantly reduce liver necrosis, especially in the high-dose group (HIRI + 50 mg/kg SEN). Meanwhile, we found that HIRI modeling significantly induced a large number of lipid droplets in the liver, while sinensetin pretreatment significantly reduced HIRI-induced lipid deposition of mice in a dose-dependent manner (Figure 2B).

Besides, we further evaluate the protective effect of sinensetin in HIRI-induced liver injury from gene expression levels. The results indicated that several genes related to liver pathological and fibrosis processes such as αSMA, MMP9 and CYP540-3A41 were significantly increased in the HIRI model, while sinensetin greatly reduced the relative mRNA levels of these genes under the HIRI conditions in a concentration-dependent manner (Figure 2C). In addition, immune cell infiltration and inflammatory response are another indicators of liver injury, so we further examined the expression of inflammatory cytokines under sinensetin pretreatment. Our results suggested that the mRNA levels of inflammatory cytokines including *TNF-α*, *IL-6* and *TGF-β* were remarkably upregulated in HIRI-induced liver injury, however, the expression of which exhibited a concentration dependent decrease under HIRI + L-SEN and HIRI + H-SEN conditions (Figure 2D). Collectively, these findings confirmed that cordycepin pretreatment alleviated HIRI-induced liver damage and systemic inflammatory response.

### 3.3 Sinensetin significantly reduced HIRI-induced liver apoptosis and oxidative stress

**FIGURE 3:**
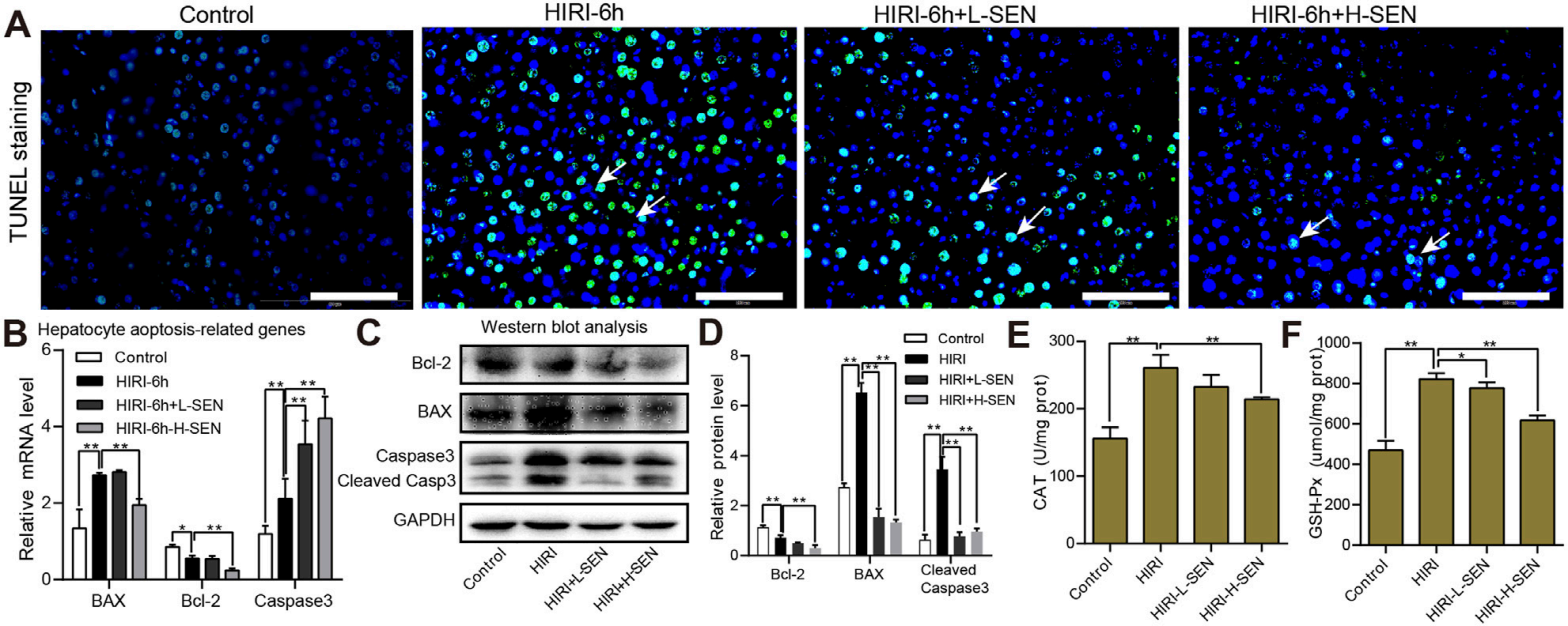
Sinensetin pretreatment significantly reduced HIRI-induced hepatocyte apoptosis and oxidative stress. **(A)** Representative image of hepatocytic apoptosis by TUNEL staining in each group. The white arrow indicated the area where apoptotic cells were located. Scale bar = 200 µm. **(B)** The relative mRNA expression levels of apoptosis-related genes were analyzed by using qRT-PCR experiments in each group. **(C)** The protein expression levels of Bcl-2, BAX and cleaved Caspase3 in mouse liver tissues were detected by Western blot analysis. **(D)** Quantitation of the protein expression of Bcl-2, BAX and cleaved Caspase3 in each group by ImageJ software. **(E)** The antioxidant enzyme activities of catalase (CAT) were measured in each group (n = 4). **(F)** The antioxidant enzyme activities of glutathione peroxidase (GSH-Px) were detected in each group (n = 4). Values are presented as mean ± SEM. **p < 0.05, **p < 0.01 vs. the HIRI group.

To further investigate the effect of sinensetin pretreatment on HIRI model, we analyzed the cell apoptosis of liver tissues in HIRI and sinensetin-treated group by TUNEL staining. TUNEL is a widely recognized indicator of apoptosis, and our findings indicated that the number of apoptotic liver cells was significantly greater in the HIRI-6h model group than in the control group, while 50 mg/kg sinensetin pretreatment (HIRI + H-SEN) could significantly reduce the level of liver cell apoptosis (Figure 3A). Simultaneously, we assessed the relative mRNA and protein expression levels of apoptosis genes in each group. The findings indicated a significant increase in the mRNA levels of pro-apoptotic genes BAX and Caspase3 in HIRI group, whereas sinensetin pretreatment could considerably decrease the expression of these genes (Figure 3B). Furthermore, Western blot analysis suggested that BAX and cleaved Caspase3 were also increased in HIRI group, while sinensetin pretreatment could significantly reduce the expression of these genes but the anti-apoptotic gene Bcl-2 showing an opposite expression trend (Figures 3C, D).

On the other hand, there is a close relationship between oxidative stress and liver damage, and these oxidative free radicals such as reactive oxygen species (ROS) could damage cell structure and function. We also further analyzed the antioxidant enzyme activities after HIRI and sinensetin pretreatment in mice. The results suggested that the enzyme activities of CAT in remarkably increased in the HIRI group while greatly decreased in the HIRI + H-SEN conditions (Figure 3E). Similarly, the antioxidant activities of GSH-Px was significantly improved in the HIRI model but notably reduced under the HIRI + sinensetin conditions in a dose-dependent manner (Figure 3F). Taken together, these results demonstrated that sinensetin plays an important protective role in hepatocyte apoptosis and oxidative stress in mice.

### 3.4 Sinensetin alleviated the HIRI-induced injury by regulating ER stress

**FIGURE 4:**
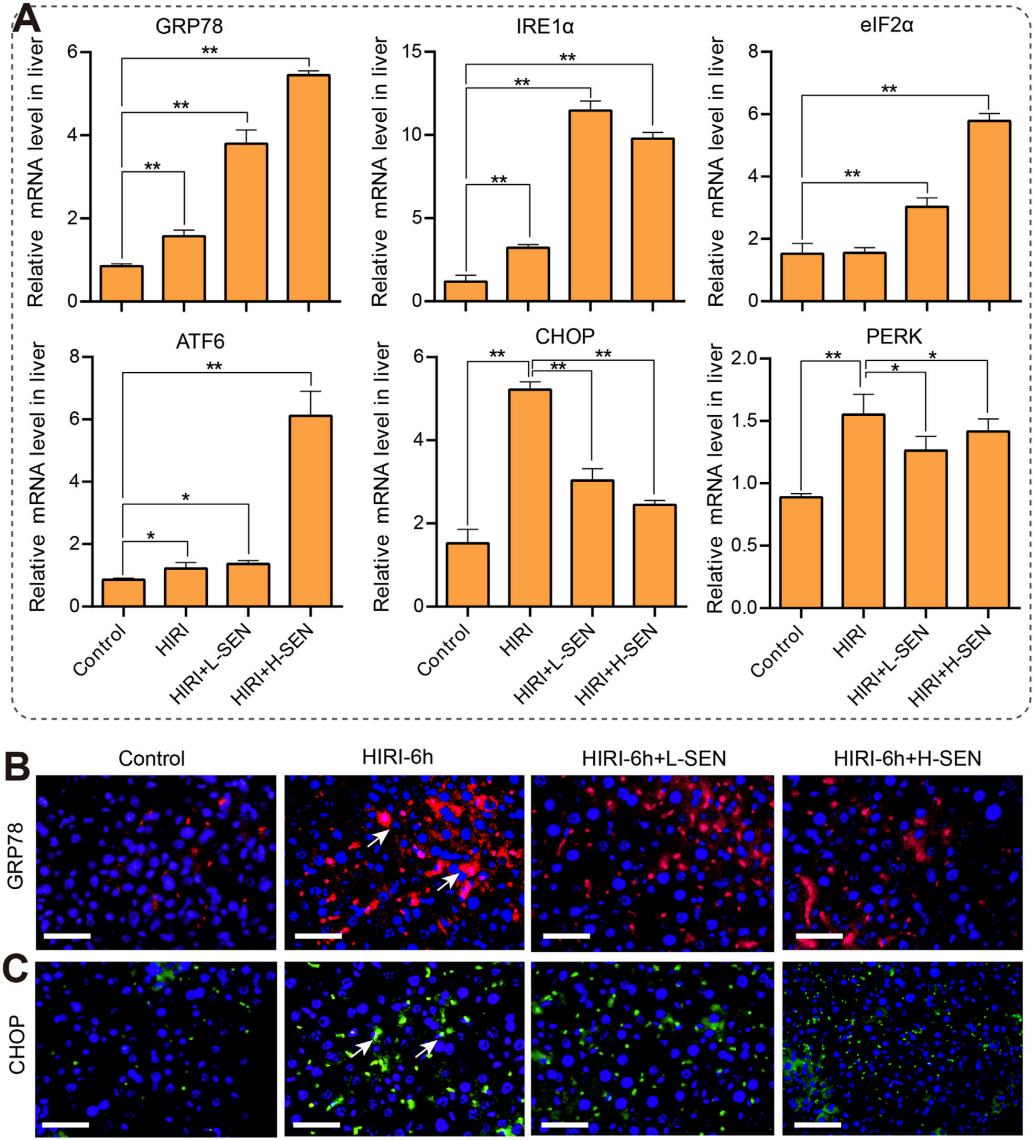
Sinensetin attenuated in HIRI-induced liver injury by regulating the endoplasmic reticulum (ER) stress signaling pathway. **(A)** The mRNA levels of ER stress markers genes were detected in each group by quantitative real-time PCR assay (n = 4). Data was shown as mean ± SEM of representative experiments; *p < 0.05, **p < 0.01 vs. the HIRI group. **(B)** The representative images of GRP78 immunofluorescence staining (red) that was co-stained with DAPI (blue) in each group. **(C)** The immunofluorescence staining of CHOP (green) was co-stained with DAPI (blue) in each group. Scale bar = 200 μm.

Endoplasmic reticulum (ER) stress signaling plays a role in heart, liver and lung injury. Therefore, we attempted to investigate whether key genes in the ER signaling pathway play a role in HIRI-induced liver injury by real time quantitative PCR analysis and the results were shown in Figure 4A. GRP78 is a glucose regulated protein, also known as immunoglobulin heavy chain binding protein (BIP), which could assist in the correct folding proteins and maintain the stability of the endoplasmic reticulum. Our results revealed that the mRNA levels of GRP78 was significantly increased on the HIRI model, and SEN pretreatment can lead to a gradient-dependent increase in the expression of GRP78. Similarly, IRE1α (inosital-requiring enzyme-1) regulates protein folding status within the endoplasmic reticulum or participates in unfolded protein reactions, the expression of which was also significantly increased in the HIRI group, and pretreatment with SEN can lead to further elevation of IRE1α expression. In addition, eIF2α and ATF6 are important proteins in ER stress response, participating in cell protection and apoptosis processes through different signaling pathways. The results suggested that eIF2α gene was significantly increased in both SEN pretreatment groups, while ATF6 was significantly increased in all treatment groups, especially in the sinensetin high concentrationgroup (50 mg/kg SEN +HIRI). It is worth mentioning that CHOP and PERK, two ER stress marker genes, were significantly elevated in the HIRI group, while they showed a significant decrease in both SEN pretreatment groups.

To enhance our understanding of the molecular mechanisms involved in ER stress during ischemia-reperfusion injury, we conducted an analysis of the expression and distribution patterns of two crucial ER stress proteins within liver tissues using immunofluorescence staining. Our findings revealed a notable increase in GRP78 protein in the HIRI-6h treatment group. In comparison to the control group, both SEN pretreatment groups also exhibited significant rises in protein expression, although these increases were not as pronounced as those observed in the HIRI group alone (Figure 4B). Compared with the HIRI model group, high concentration pretreatment with SEN significantly reduced the expression and distribution of CHOP protein in liver tissue (Figure 4C). Overall, ER stress signaling is involved in the protective mechanism of SEN against HIRI-induced liver injury in mice.

### 3.5 ER proteins was involved in the protective effect of SEN in HIRI-induced liver injury

**FIGURE 5:**
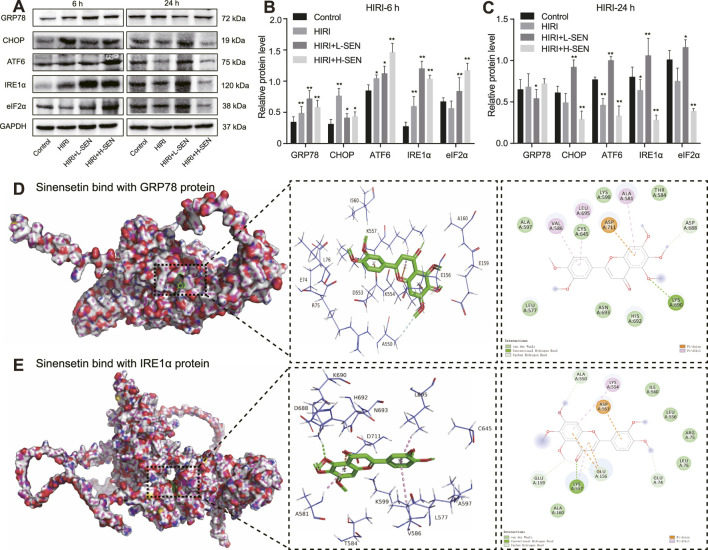
Sinensetin plays a protective role in HIRI-induced liver injury through the interaction of ER stress marker proteins. **(A)** Western blot images exhibiting ER stress marker protein levels of GRP78, CHOP, ATF6, IRE1α and eIF2α in reperfusion for 6 h and 24 h, respectively. **(B)** Quantitative analysis of ER stress marker protein levels in reperfusion for 6 h by ImageJ software. **(C)** Quantitative intensity of ER stress marker protein in reperfusion for 24 h by ImageJ software. Data are presented as mean ± SEM (n = 4 in each group). **p < 0.05 and **p < 0.01 vs. the control group. **(D)** The molecular docking results of sinensetin and GRP78 protein. The docking position of sinensetin and GRP78 were performed with the AutoDock Vina and visualized with the PyMOL software. **(E)** The molecular docking model of sinensetin and IRE1α. The optimal binding position were displayed in the left panel and the enlarged 3D diagram and 2D diagram of molecular docking were presented in the right panel. The dashed line represented the distance between hydrogen bonds.

Next, we detected the expression of ER stress-related proteins at 6 and 24 h after perfusion by Western blot analysis. The results indicated that the HIRI-model could induce an significant increase in the expression of majority proteins such as CHOP, GRP78 and IRE1α after 6 h of perfusion. In addition, sinensetin pretreatment can further improvement the expression of ER stress proteins such as GRP78, ATF6, IRE1α and eIF2α (Figures 5A, B). However, after 24 h of perfusion, these proteins decreased to lower levels than the normal control group in both the HIRI model group and the HIRI + H-SEN group. Interestingly, the expression of these ER stress proteins remained at higher levels in the HIRI + L-SEN group (Figures 5A, C). Overall, these results indicated that the endoplasmic reticulum stress signaling pathway mainly played an important role in the early stages of liver ischemia-perfusion in mice, and the expression of these key genes and ER proteins presented different expression characteristics.

In order to further understand the interaction between SEN and ER stress proteins in HIRI-induced liver injury, we simulated the theoretical possibility of SEN and GRP78, as well as SEN and IRE1α proteins, binding to each other in spatial structure through molecular docking analysis. Our results revealed that sinensetin and GRP78 proteins formed a stable three-dimensional structure with a binding energy of −5.2 kcal/mol. The interaction involved specific amino acid residues including GLU-156, ASP-553, LYS-554 andLYS-557, which formed multiple hydrogen bonds (Figure 5D). In addition, our findings revealed that SEN and IRE1α proteins had a higher binding energy of −7.4 kcal/mol. The interaction involved specific amino acid residues such as ALA-581, CYS-645, LYS-690, LEU-695 and ASP-711 that formed stable hydrogen bonds (Figure 5E). These results further demonstrated that sinensetin and GRP78/IRE1α exhibited strong binding properties and may possible involvement in the protective mechanism of sinensetin in HIRI-induced liver injury in mice.

### 3.6 Pharmacological inhibition of ER stress can alleviate HIRI-induced liver injury

**FIGURE 6:**
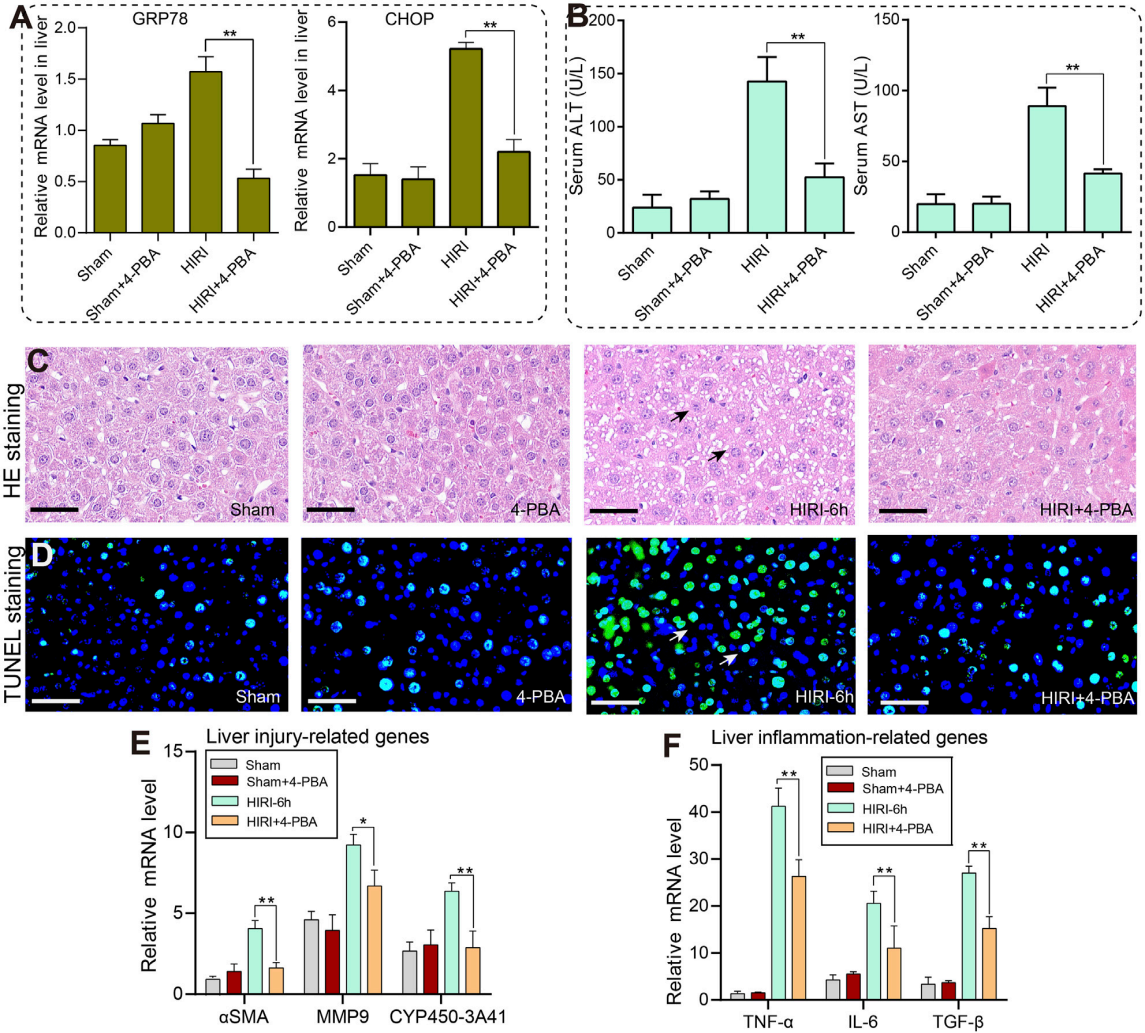
Inhibition of ER stress with 4-PBA can significantly improve liver injury caused by ischemia-reperfusion in mice. **(A)** The relative mRNA levels of GRP78 and CHOP genes in the sham, 4-PBA (20 mg/kg), HIRI-6h, and HIRI-6h+4-PBA (20 mg/kg) groups. **(B)** The serum ALT and AST levels were detected in each group. **(C)** The histopathological features of the liver were observed in each group by H&E staining. Black arrows indicated the area of liver cell necrosis. **(D)** The cell apoptosis of liver tissues in each group were detected by TUNEL staining. The white arrow marks the area where apoptotic cells are located. **(E)** The mRNA expression levels of liver injury-related genes were measured by qRT-PCRs in each group. **(F)** The mRNA expression levels of liver inflammation-related genes were analyzed by qRT-PCRs in each group. The results were obtained from at least three independent experiments. Values are presented as mean ± SEM. **p < 0.05, **p < 0.01 vs. the HIRI-6h group.

Next, we conducted pharmacological experiments to investigate whether endoplasmic reticulum stress signaling is involved in HIRI-induced liver injury in mice. 4-Phenylbutyric acid (4-PBA) is a ER stress inhibitor, and we found that 20 mg/kg 4-PBA pretreatment could significantly reduce the mRNA expression level of GRP78 and CHOP, respectively (Figure 6A). In addition, compared with the HIRI group alone, there was a significant decrease in serum ALT and AST levels in the HIRI+4-PBA group (Figure 6B). On the other hand, we further analyzed whether 4-PBA pretreatment can rescue the phenotype of liver injury in mice. H&E staining results showed that there was not much difference between 4-PBA treatment alone and the sham group, but the HIRI+4PBA co-treatment group significantly reduced the number of liver cell necrosis compared to the HIRI group alone (Figure 6C).

**FIGURE 7:**
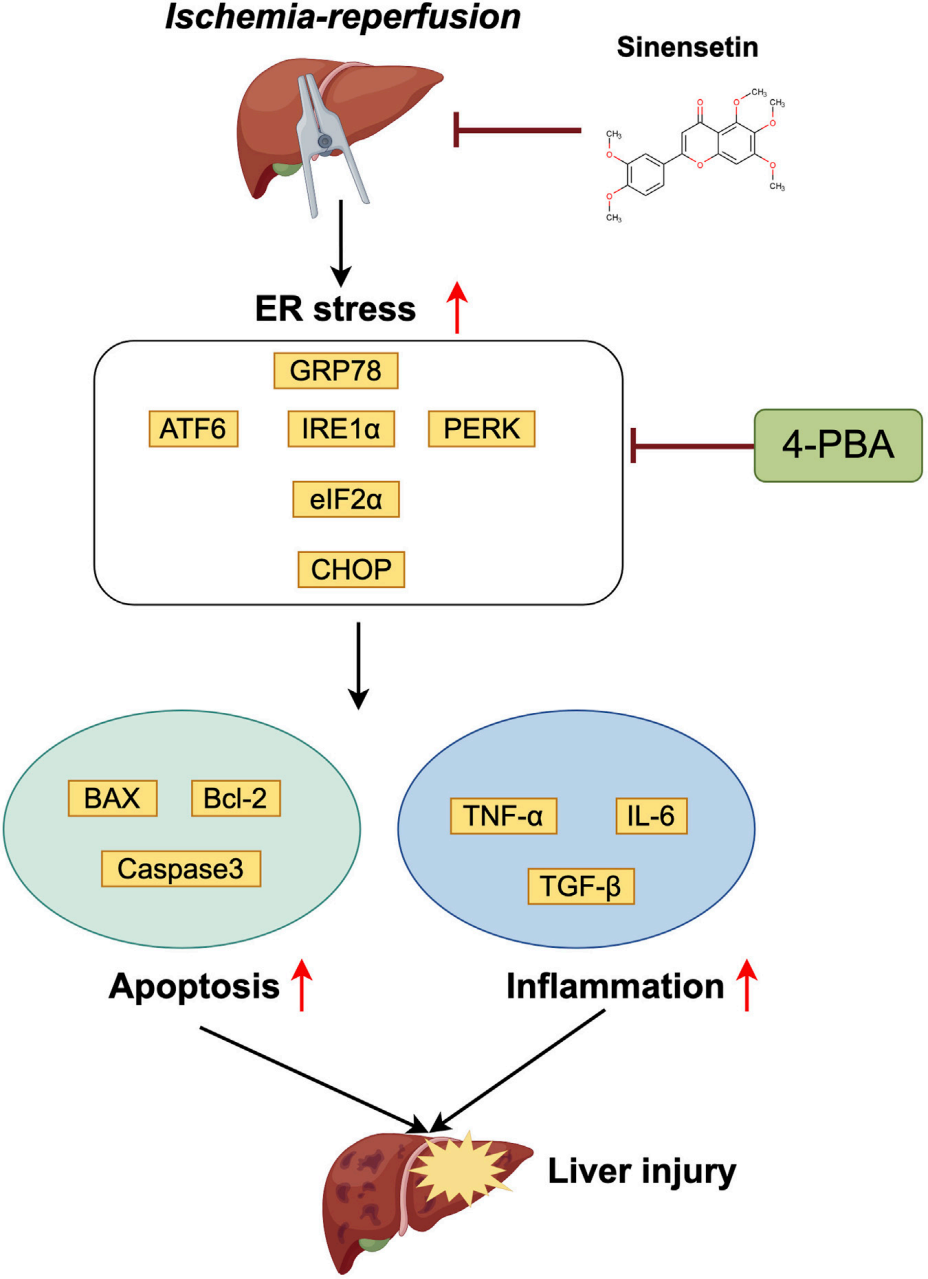
Schematic representation of the liver protective effect of sinensetin in the hepatic schemia-reperfusion injury in mice.

Furthermore, TUNEL staining revealed a significant increase in liver apoptosis levels in the HIRI group. However, pretreatment with the 4-PBA inhibitor partially rescued and alleviated the apoptotic levels of liver tissues under the ischemia-reperfusion conditions (Figure 6D). Finally, we observed a significant decrease in the expression of liver injury-related genes such as αSMA, MMP9 and CYP450 under the co-exposure of HIRI-6h+4-PBA inhibitor conditions compared with the HIRI group (Figure 6E). Meanwhile, the inflammatory cytokine such as TNF-α, IL-6 and TGF-β were all significantly downregulated in the HIRI+4-PBA group compared with the HIRI group (Figure 6F). Based on the results mentioned above, a schematic model of the molecular mechanisms involved in this study was presented in Figure 7. In conclusion, our data demonstrate that ER stress signaling was involved in HIRI-induced liver injury and played an important role in the liver protective effect of sinensetin in mice.

## 4 Discussion

Hepatic ischemia-reperfusion injury (HIRI) often arises from surgical interventions like hepatectomy and liver transplantation (Zhang et al., 2021; Ma et al., 2023). This pathophysiological condition worsens during the reperfusion phase following hepatic ischemia (Montalvo-Jave et al., 2008). Post-reperfusion, the damage to liver tissue is linked to inflammatory responses, hepatocyte apoptosis, oxidative stress, and endoplasmic reticulum stress, which can significantly impair patient prognosis and leading to liver injury and organ dysfunction (Liu and Man, 2021). Currently, it is crucial to investigate the molecular mechanisms involved to discover potential preventive and therapeutic strategies for HIRI. Sinensetin is a polymethoxylated flavonoid primarily derived from citrus fruits and exhibits anti-inflammatory, antioxidant and immunomodulatory properties (Han Jie et al., 2021). Nevertheless, the hepatoprotective effects of sinensetin in ischemia-reperfusion injury remain unclear. Therefore, a mouse model of HIRI was developed to assess whether sinensetin can provide protective effect against HIRI and to clarify the underlying mechanisms involved.

After 60 min of ischemia, we found a significant whitening of the left liver lobe, indicating successful HIRI modeling in mice. The levels of certain enzymes, including ALT, AST, and LDH, are acknowledged as effective markers for assessing the extent of liver injury (Church et al., 2019). In this study, notable increases in the serum concentrations of ALT and AST were detected at both 6 h and 24 h following reperfusion, which indicating severe liver injury occurring following reperfusion. It is worth mentioning that the activity of these enzymes increased at most at HIRI-6 h compared to HIRI-24 h, implying that the early stages of liver injury are particularly critical. Besides, pretreatment with sinensetin resulted in a notable reduction in liver enzyme activity in a dose-dependent manner. This finding was further supported by pathological assessments and indicated that sinensetin substantially reduced the degree of liver necrosis and lipid accumulation in mice. The previous studies, serum ALT and AST levels were notable increased in the HIRI group, while these changes were partly reversed by octreotide treatment indicating that the liver injury triggered by HIR was partially attenuated by octreotide (Zou et al., 2022). Sodium nitrite reduced HIRI in rats and resulted in a constrained increase in serum levels of ALT and AST caused by liver I/R damage (Choi et al., 2018). In accordance with the previous studies, we also confirmed the hepatoprotective effect of sinensetin in liver I/R injury of mice. Although there are currently many drugs that can improve liver ischemia-reperfusion injury, such as Octreotide, Melatonin and Cordycepin (Gao et al., 2021; Ding et al., 2022; Zou et al., 2022). We have demonstrated for the first time that sinensetin extracted from citrus fruits have attenuated the HIRI effects while having smaller negative effects.

The αSMA, MMP9 and CYP450-3A41 genes have unique functions in liver injury, and our results have shown that sinensetin can significantly reduce the increased expression of these genes induced by HIRI in mice. Meanwhile, sinensetin reduced the expressions of TNF-α, IL-6 and TGF-β in a dose-dependent manner, indicating that SEN can protect the liver by reducing the release of inflammatory mediators. Apoptosis of liver cells is a complex process involving the regulation of multiple genes, mainly involving promoting apoptotic genes such as Bax and inhibiting apoptotic genes such as Bcl-2 and Caspase family genes (Chu et al., 2021). In this study, we observed a significant decrease in the number of TUNEL-positive cells in the groups that received sinensetin pretreatment, as indicated by TUNEL staining. An analysis of apoptosis-related genes revealed that pretreatment with sinensetin resulted in lower expression levels of BAX and Bcl-2, while promoting an increase in the expression of Caspase3. From the results of antioxidant enzyme activity detection, high concentration SEN pretreatment also led to a significant decrease in HIRI-induced injury including CAT and GSH-Px enzyme activity levels. In consistent with previous studies, the extent of liver injury was significantly attenuated in mice that were orally administered with tea polyphenols (TP), which the I/R-induced liver cell apoptosis is inhibited by TP pretreatment (Tao et al., 2016).

In recent years, ER stress is a newly identified pathway significantly contributing to liver I/R injury (Zhu and Zhou, 2021). ER stress can induce cell apoptosis, regulate unfolded protein response and activate inflammatory response (Xu et al., 2015). Our results indicated that the key genes of the ER signaling pathway were dynamically regulated during HIRI and SEN pretreatment in qRT-PCR analysis. Immuno-fluorescence and Western blot experiments showed that GRP78 and CHOP proteins were significantly increased under HIRI conditions, while HIRI + SEN treatment could lead to a significant decrease in them. At the same time, the expression levels of other proteins such as ATF6 and IRE1α were further increased under SEN pretreatment conditions. Moreover, molecular docking analysis showed theoretical binding between SEN-GRP78 and SEN-IRE1α proteins in spatial structure, suggesting that sinensetin may play a role in resisting HIRI-induced liver injury by regulating ER stress signaling pathway. In accordance with previous studies, rapamycin markedly protected livers from HIRI and ER stress markers were markedly upregulated by HIRI treatment, whereas they were downregulated by rapamycin pretreatment (Zhu et al., 2015). Ischemic preconditioning attenuates endoplasmic reticulum stress-dependent apoptosis of hepatocytes in liver I/R injury and hydrogen-rich saline ameliorates HIRI through regulation of ER stress and apoptosis (Lu et al., 2017; Kong et al., 2023).

Based on the previous studies, we revealed that ER stress may be involved in the liver damage induced by HIRI in mice. Therefore, we aimed to investigate the role of the GRP78/IRE1α signaling pathway in the liver injury caused by HIRI. Our results demonstrated that pharmacological inhibiting the ER stress signaling by 4-PBA pretreatment could decrease the expressions of GRP78 and CHOP, together with significantly reduce the key liver function indicator ALT and AST levels. Meanwhile, 4-PBA pretreatment partially ameliorated the degree of hepatocyte necrosis and liver apoptosis induced by HIRI in mice. Finally, the expression of liver injury and inflammatory related genes also partially rescued after inhibiting ER stress, exhibiting liver protective effect similar to sinensetin treatment. In conclusion, sinensetin presents a promising therapeutic avenue for protecting against the liver from ischemia/reperfusion injury. This effect may be derived from its anti-inflammatory and anti-apoptotic properties, as well as the regulation of GRP78/IRE1α ER stress signaling pathway. Further research is needed to comprehensively clarify its mechanisms of action and explore its potential clinical applications.

## 5 Conclusion

In summary, the current study proved that sinensetin could ameliorate liver apoptosis, inflammatory response and histopathological damage following hepatic schemia-reperfusion injury I/R injury in mice. The molecular mechanism of this hepatoprotective effect may be related to the inhibition of ER stress through targeting the GRP78/CHOP proteins. Our results provided experimental evidence that sinensetin may be a potential candidate for the treatment of hepatic schemia-reperfusion injury in clinic practice.

## Data Availability

The original contributions presented in the study are included in the article/Supplementary Material, further inquiries can be directed to the corresponding authors.
