# Supplementary material for: Sinensetin attenuates hepatic ischemia-reperfusion injury through suppressing GRP78/CHOP-mediated endoplasmic reticulum (ER) stress in mice
Source: Front Pharmacol. 2025 Feb 12;16:1519497. doi: 10.3389/fphar.2025.1519497 (PMC11861360; doi:10.3389/fphar.2025.1519497)
Supplement: Supplementary file 2 [file DataSheet1.pdf]

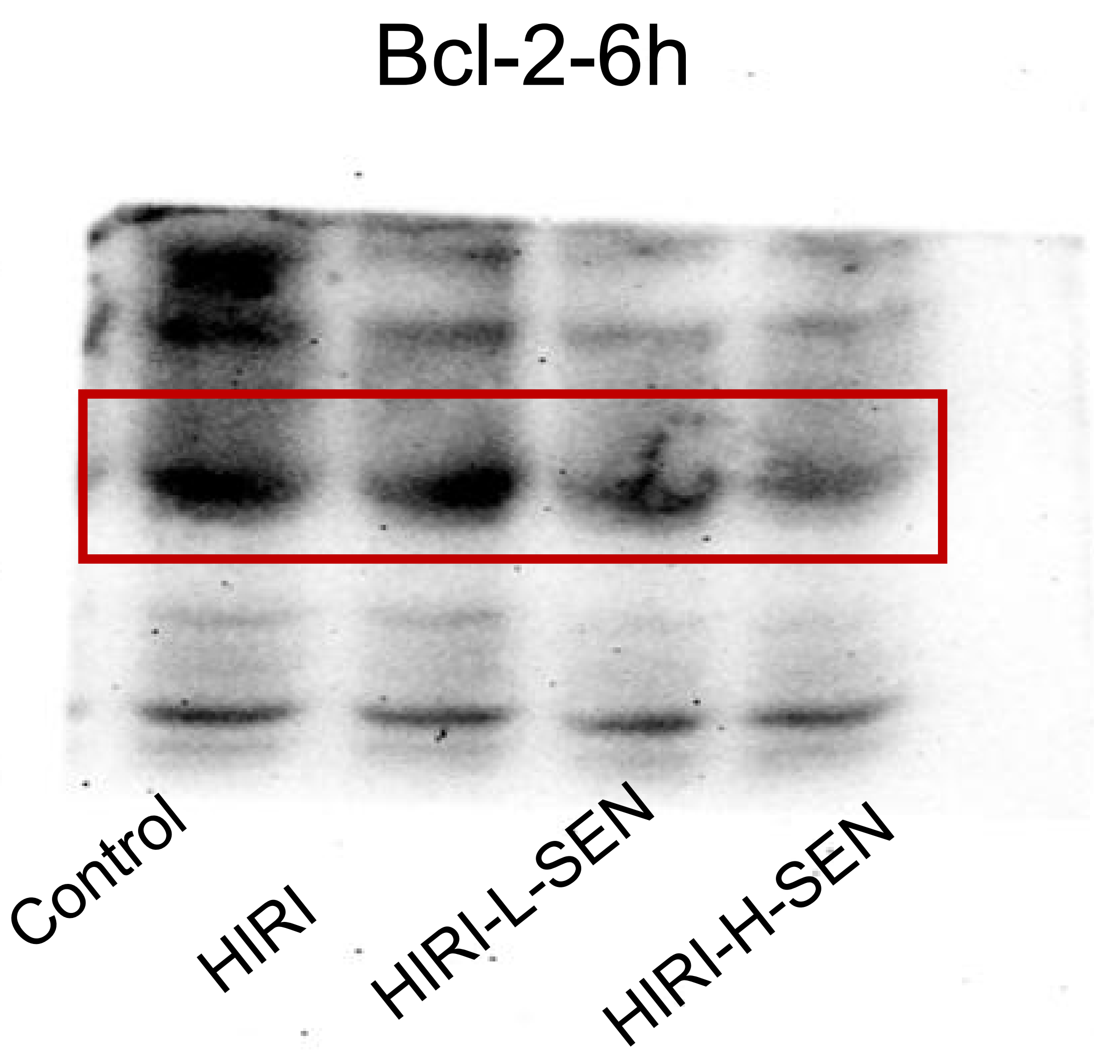
Bcl-2-6h
Control
HIRI
HIRI-L-SEN
HIRI-H-SEN

BAX-6h

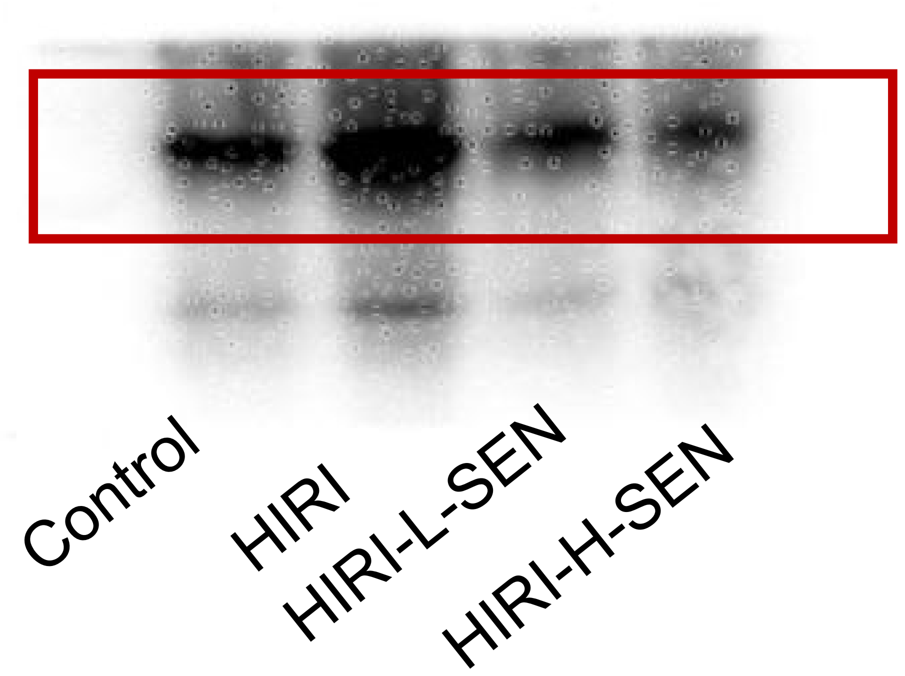

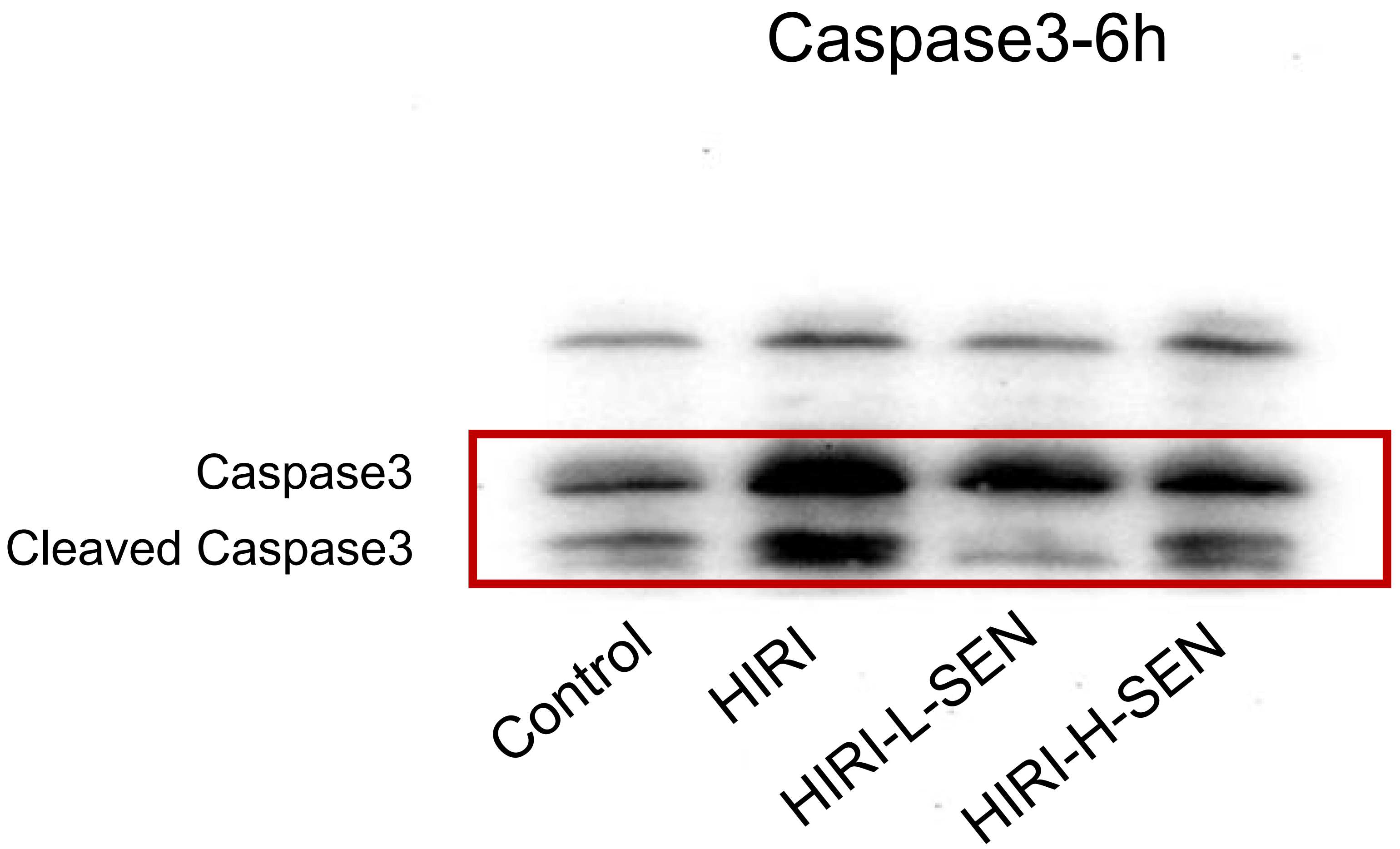
Caspase3-6h
Caspase3
Cleaved Caspase3
Control
HIRI
HIRI-L-SEN
HIRI-H-SEN

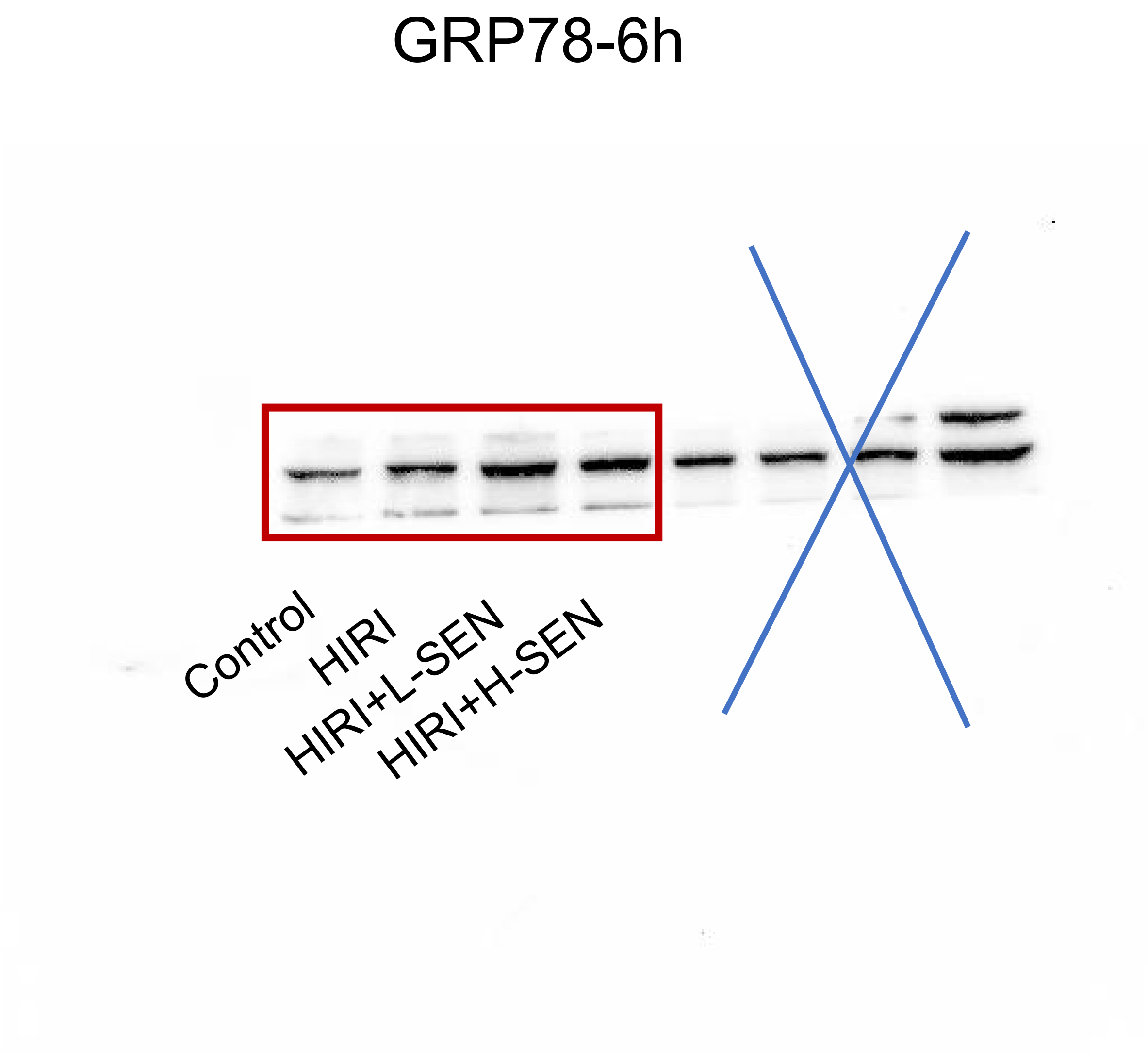
GRP78-6h
Control
HIRI
HIRI+L-SEN
HIRI+H-SEN

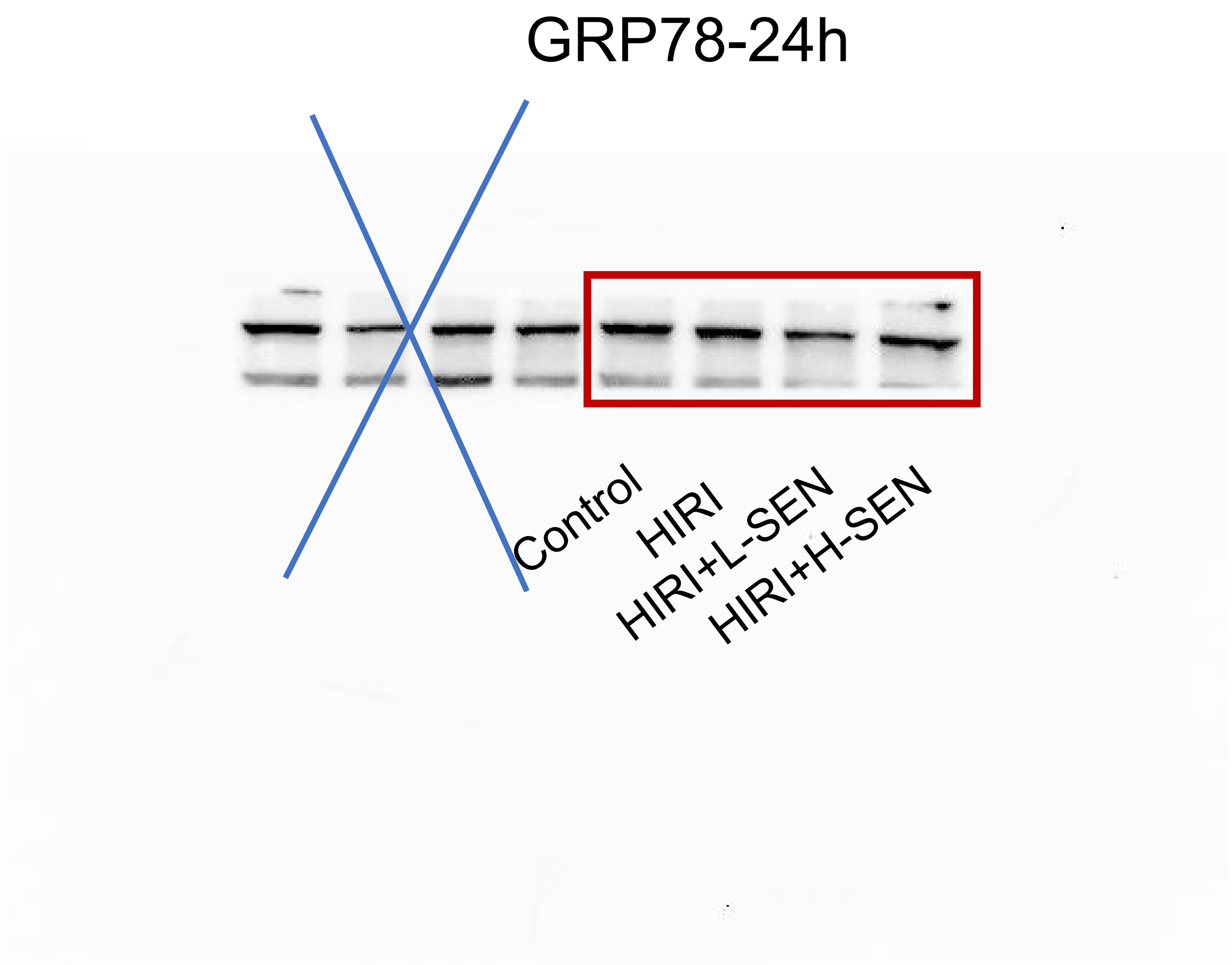
GRP78-24h
Control
HIRI
HIRI+L-SEN
HIRI+H-SEN

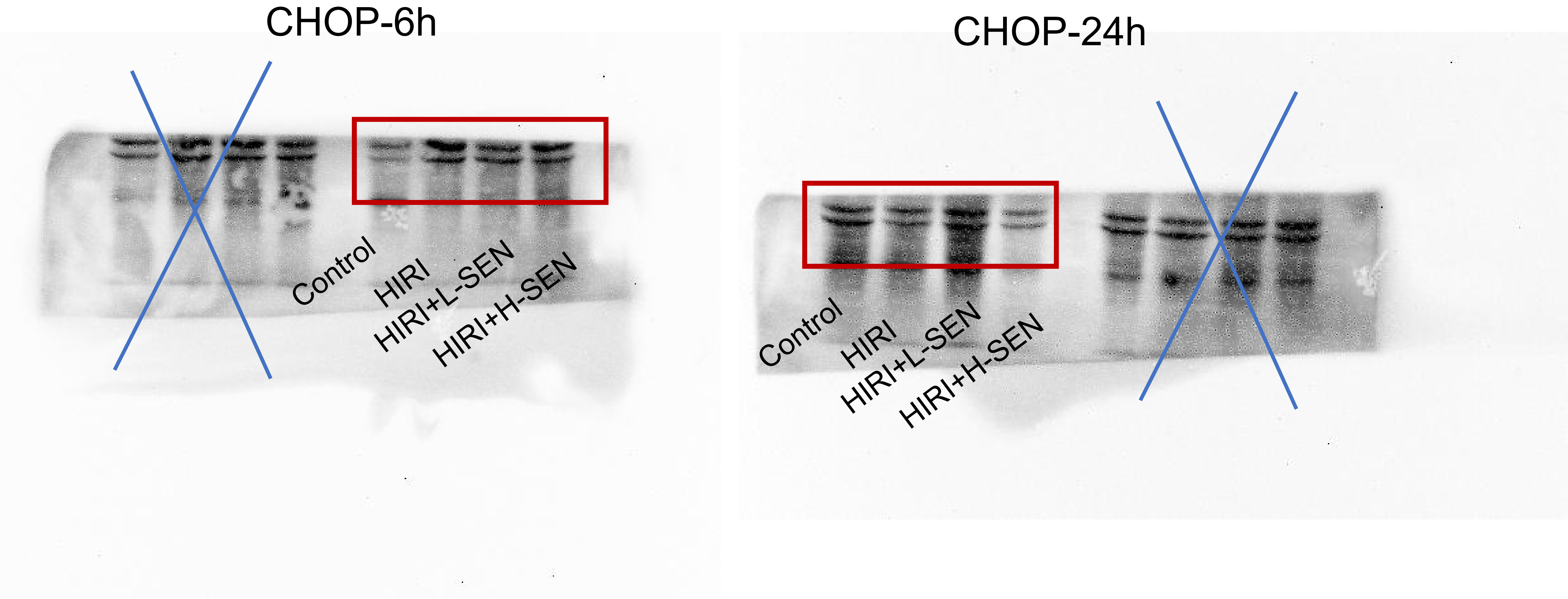
CHOP-6h
Control
HIRI
HIRI+L-SEN
HIRI+H-SEN
CHOP-24h
Control
HIRI
HIRI+L-SEN
HIRI+H-SEN

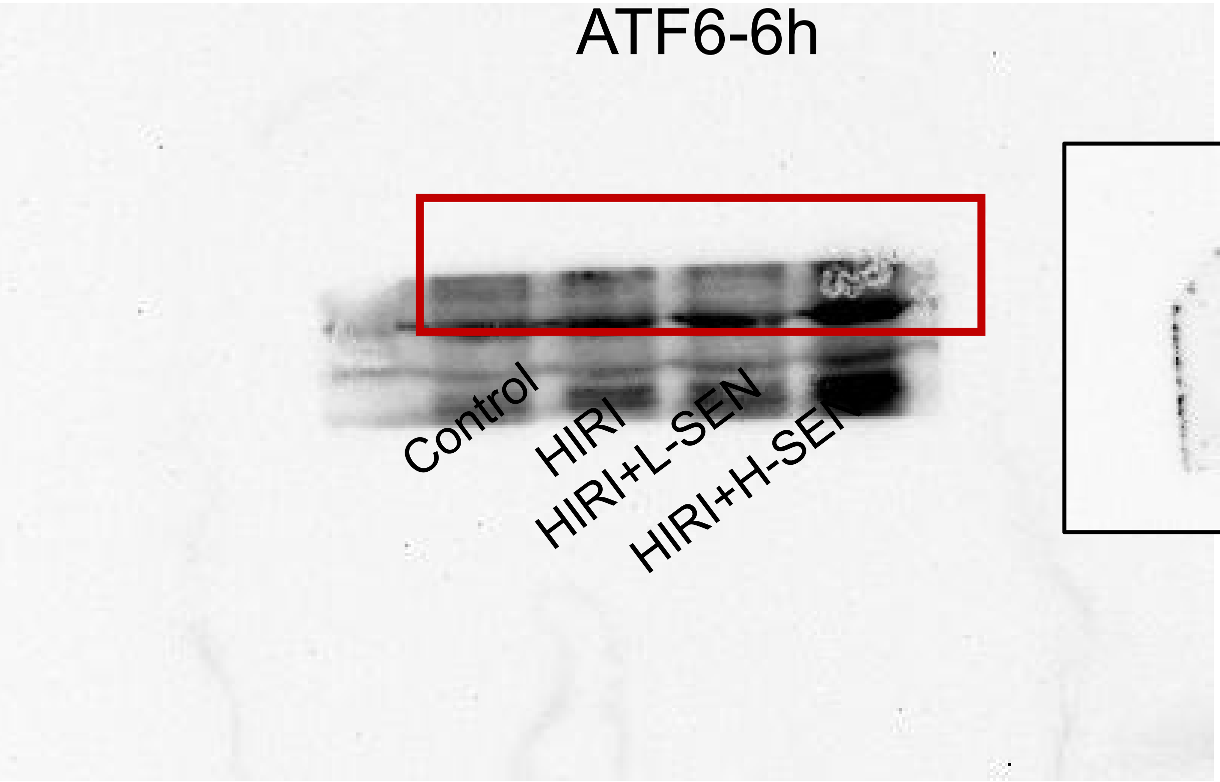
ATF6-6h
Control
HIRI
HIRI+L-SEN
HIRI+H-SEN

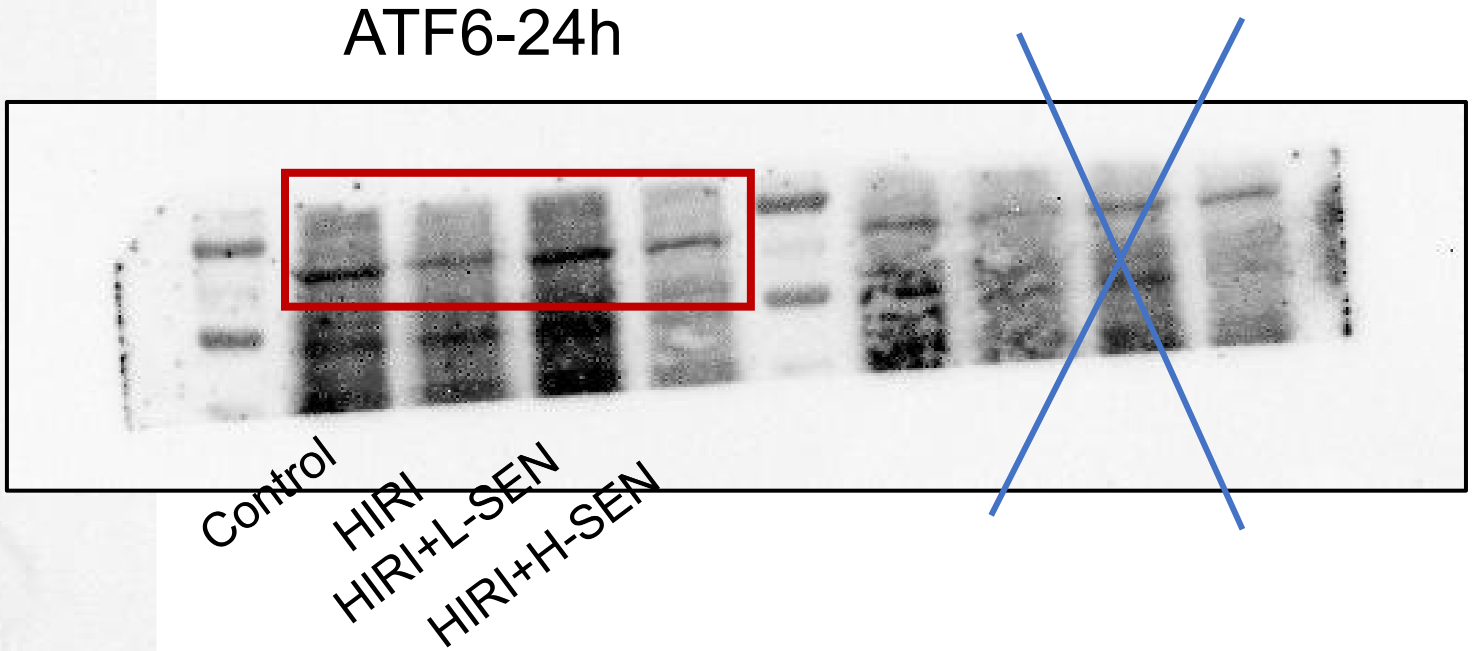
ATF6-24h
Control
HIRI
HIRI+L-SEN
HIRI+H-SEN

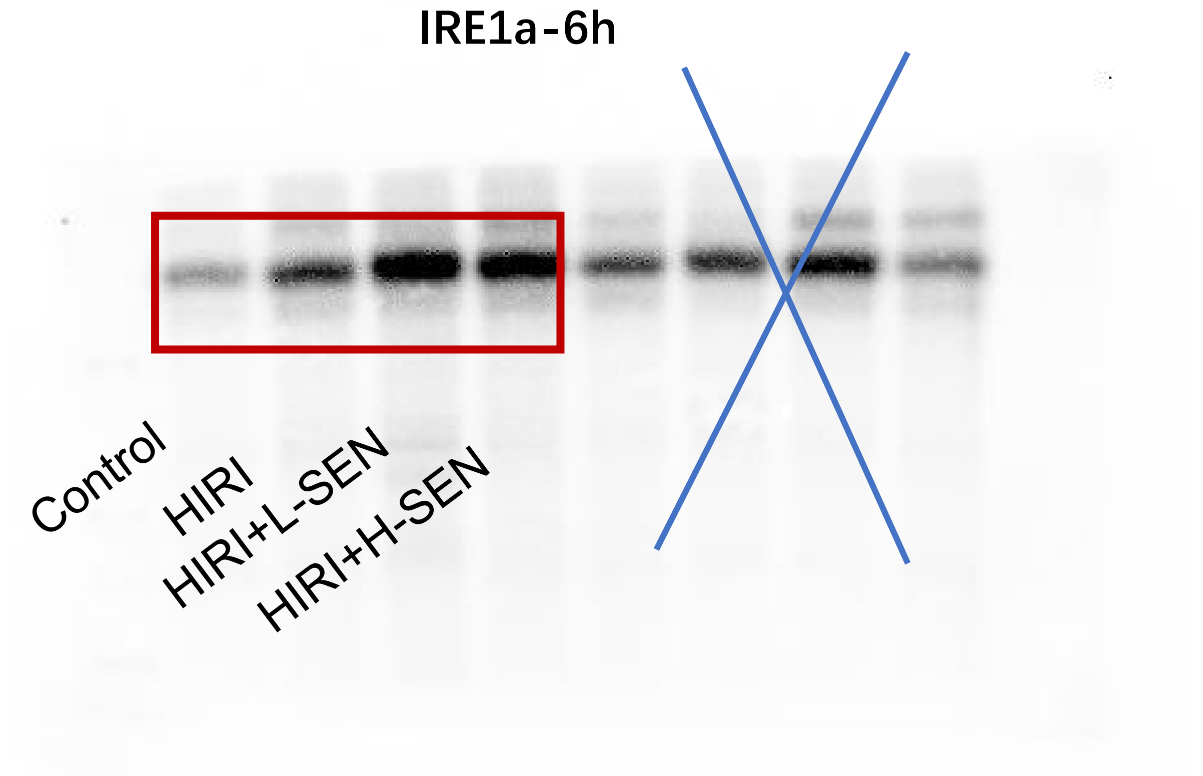
IRE1a-6h
Control
HIRI
HIRI+L-SEN
HIRI+H-SEN

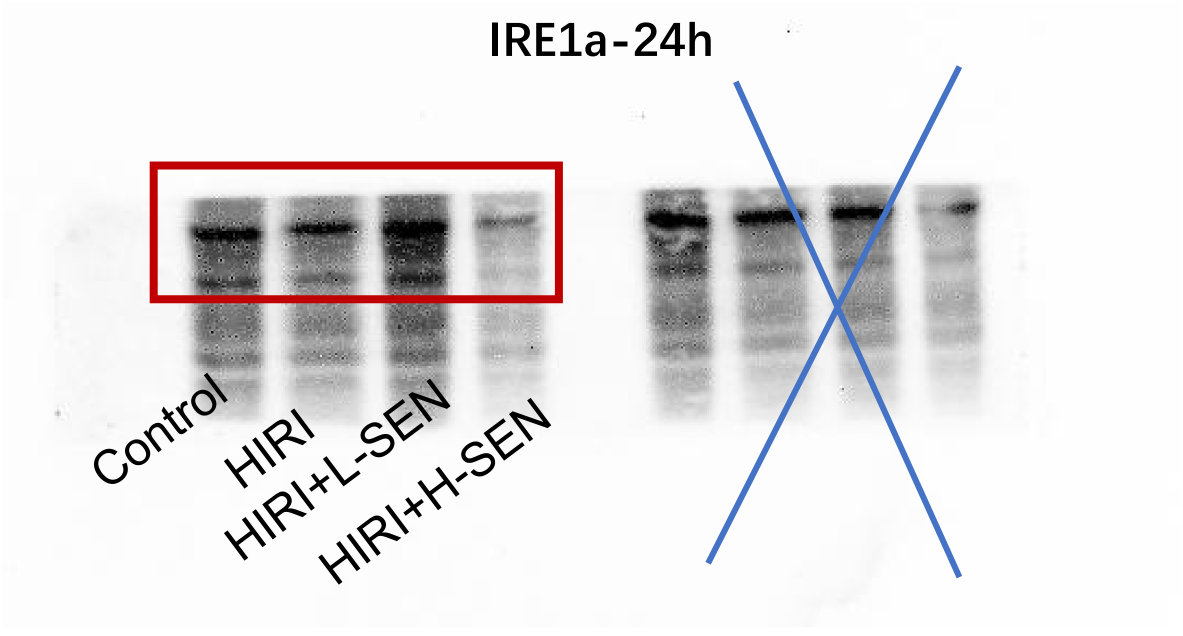
IRE1a-24h
Control
HIRI
HIRI+L-SEN
HIRI+H-SEN

eIF2a-6h

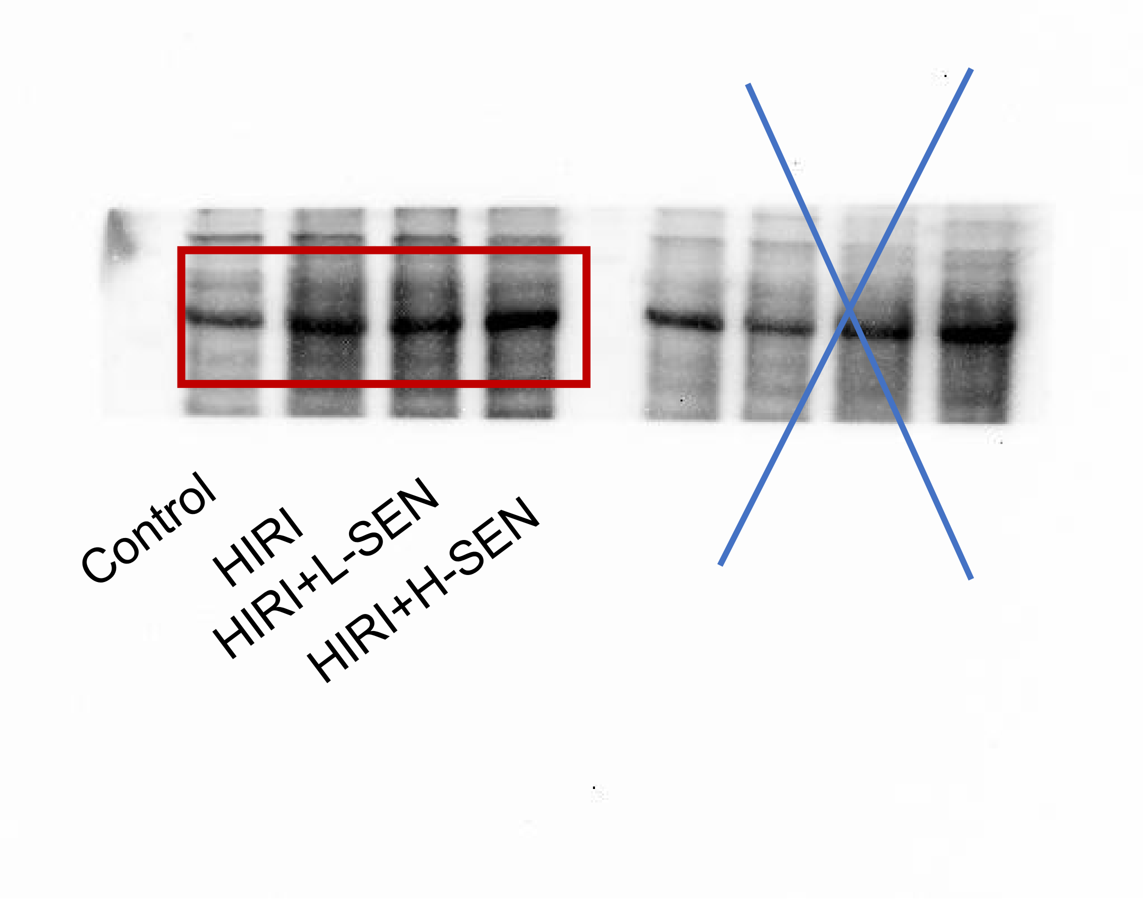

eIF2a-24h

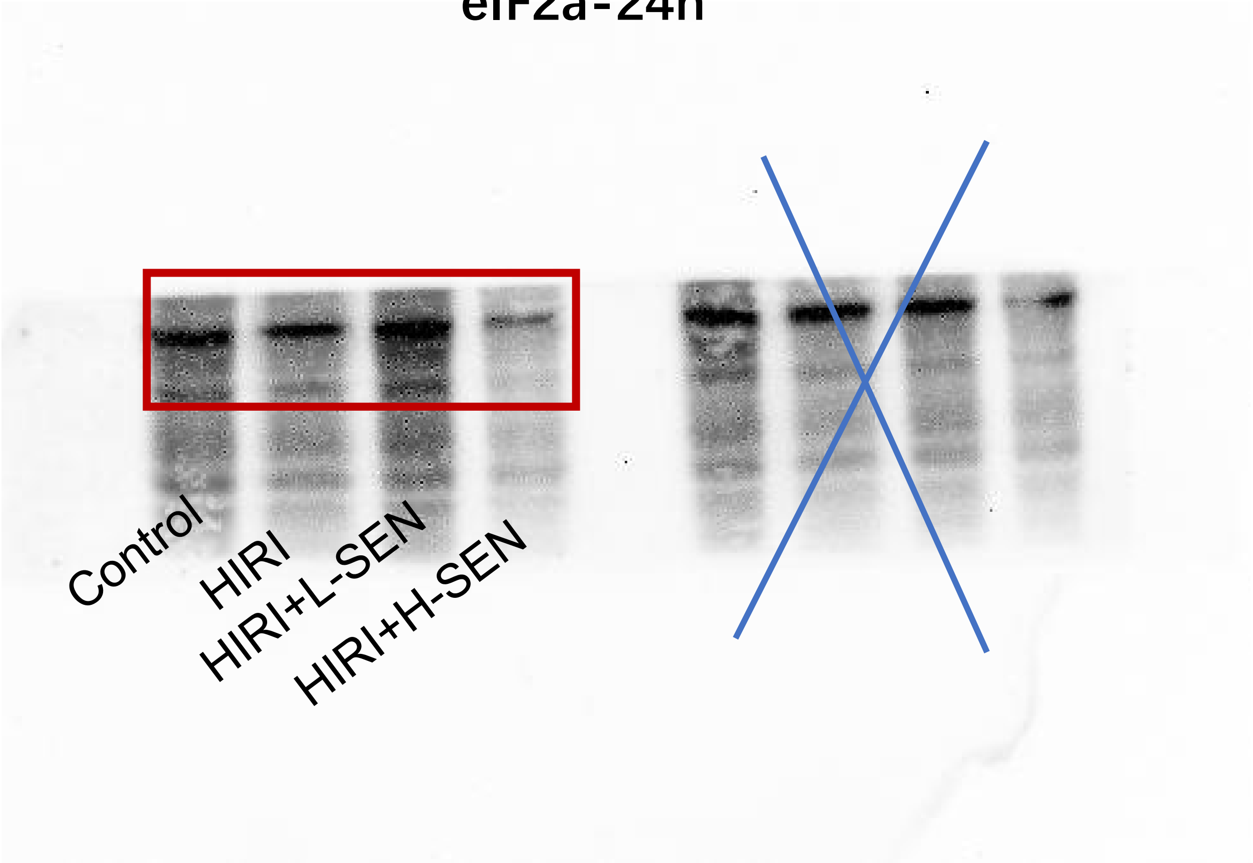

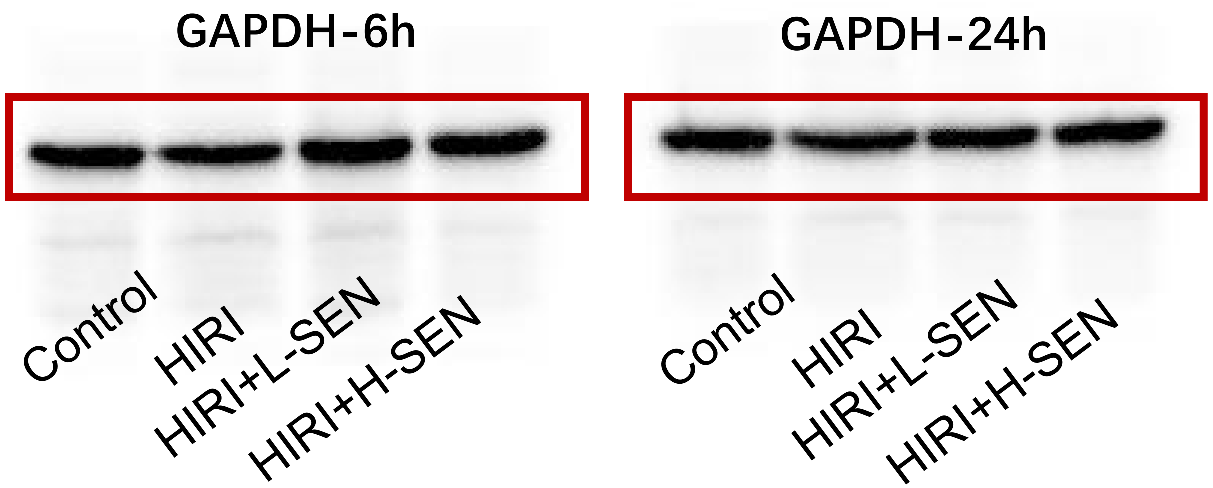
GAPDH-6h
Control
HIRI
HIRI+L-SEN
HIRI+H-SEN
GAPDH-24h
Control
HIRI
HIRI+L-SEN
HIRI+H-SEN
